# Delayed Infliximab Treatment Affects the Outcomes of Patients With Crohn's Disease During the COVID-19 Epidemic in China: A Propensity Score-Matched Analysis

**DOI:** 10.3389/fmed.2021.819557

**Published:** 2022-01-12

**Authors:** Yong Li, Lulu Chen, Shuijiao Chen, Xiaowei Liu

**Affiliations:** ^1^Department of Gastroenterology, Xiangya Hospital, Central South University, Changsha, China; ^2^Hunan International Scientific and Technological Cooperation Base of Artificial Intelligence Computer Aided Diagnosis and Treatment for Digestive Disease, Xiangya Hospital, Central South University, Changsha, China; ^3^National Clinical Research Center for Geriatric Disorders, Xiangya Hospital, Changsha, China

**Keywords:** COVID-19, infliximab, Crohn's disease, delayed treatment, readmission

## Abstract

**Background:** The coronavirus disease 2019 (COVID-19) has swept the world and led to delays in the treatment of Crohn's disease patients treated with biologics. This study aims to investigate the risk factors for delayed treatment during the epidemic and to observe the short- and long-term influences of such delays among them to provide some reference on treatments.

**Methods:** This study retrospectively enrolled patients diagnosed with Crohn's disease who received infliximab treatment between January 23, 2020 and April 30, 2020. Univariate and multivariate logistic regression were used to analyze the risk factors for delayed treatment. Propensity score matching was utilized to compare the effects of delayed treatment on the short- and long-term outcomes.

**Result:** Our cohort identified a total of 53 patients with a delay rate of 71.7%. Of these patients, 38 were in the delayed group, and 15 were in the non-delayed group. Logistic regression analysis showed that the baseline levels of C-reactive protein were an influence factor for delaying treatment (OR = 0.967, 95% CI = 0.935–1.000, *p* = 0.047). Regarding short-term effects, the delayed group had a lower decrease in the Crohn's Disease Activity Index than the non-delayed group [−43.3 (−92.7, −9.7) vs. −17.3 (−29.0, 79.9), *p* = 0.038] and significantly higher long-term readmission rates (33.3% vs. 0%, *p* = 0.014).

**Conclusion:** Delayed infliximab treatment could affect the short- and long-term outcomes in patients with Crohn's disease. Our study suggested that the regulated course of treatment with biological agents should be performed effectively and that education should be enhanced to minimize delays in treatment.

## Introduction

The coronavirus disease 2019 (COVID-19) pandemic originating from severe acute respiratory syndrome coronavirus 2 (SARS-CoV-2) was an ongoing outbreak that was more widespread than any pandemic from the past century (1). On March 11, 2020, the World Health Organization declared COVID-19 a global pandemic (2). The main route of transmission was aerosol spread by patients in the air (3).

Although COVID-19 was primarily characterized by respiratory symptoms, it also causes some gastrointestinal symptoms, such as diarrhea, nausea, vomiting and abdominal discomfort (4), which is related to angiotensin-converting enzyme 2 (ACE2) used by coronaviruses to penetrate target cells (5). The ACE2 is expressed by intestinal epithelial cells, especially in the inflamed terminal ileum and colon (6). Therefore, several gastroenterologists concerned that patients with Crohn's disease (CD) may have a higher risk for COVID-19 infection. In addition, they suggested that long-term administration of steroids and immunosuppressive agents in CD patients was also a susceptibility factor (7). According to the guidelines of the most qualified international societies and organizations for the study of inflammatory bowel disease (IBD), outpatient visits for CD patients should be postponed as well as colonoscopy and non-urgent surgery to reduce the risk of contagion (8, 9). Data were reported from an international survey with a reduction of endoscopic activities by 75–100% in the majority of IBD centers (10). Furthermore, early in the epidemic, relevant vaccines were not yet available, although current guidelines considered vaccination less impactful in patients with IBD (11, 12). These all put a tremendous strain on the mind of patients. In a series of surveys of IBD patients, the rates of anxiety and depression in them have been high as 48–62% compared to the general population, and most perceived increased vulnerability due to disease in response to the pandemic (13, 14).

Therefore, for CD patients receiving regular infliximab treatment, there is a concern that weighting the pros and cons of admission to the hospital and viral infections becomes a concerning matter. Most current studies focus on the diagnosis and management of IBD patients infected with COVID-19. A few researches showed that patients on intravenous biological treatments were more likely to stop or delay treatment (15, 16). In a recent report, ~27.7% of patients with IBD discontinued their medication during the COVID-19 epidemic, among patients of whom, 29.3% suffered aggravated conditions (17). However, these studies did not have a long-term follow-up of patients with delayed treatment. Whether delayed treatment affects the long-term outcome of the disease and the efficacy of biological agents is currently unknown.

This study aimed to determine factors associated with delayed infliximab treatment in patients with CD during the COVID-19 crisis (from January 23, 2020 to April 30, 2020) and to investigate the short- and long-term clinical consequences of delayed treatment with infliximab infusion. It would provide some reference on biologics treatments in CD patients at present given that the epidemic has normalized.

## Methods

### Patient Population

Consecutive patients with CD receiving infliximab treatment between January 23, 2020 and April 30, 2020 in the Gastroenterology Department of Xiangya Hospital of Central South University were enrolled. Patients with a diagnosis of CD according to clinical, radiological, or endoscopic evidence as suggested by the European Crohn's and Colitis Organization (ECCO) guidelines with a disease duration of more than 3 months were included. In addition, included patients aged 17–75 years should have an indication for treatment with infliximab (18). Excluded patients were allergic to or intolerant of the agent or changed to infliximab treatment during the COVID-19 epidemic. Patients lost to follow-up or without complete medical record data were not considered in our research. If combined with other drugs during the follow-up, patients would be excluded.

### Study Design

All included patients received intravenous treatment with infliximab before and during the COVID-19 epidemic. The induction treatment for infliximab was 5 mg/kg at weeks 0, 2 and 6 and maintenance was 5 mg/kg every eight weeks. There were two groups (delayed group and non-delayed group) according to whether there was a delay of infliximab infusion. Data, including demographics, serological indicators [i.e., erythrocyte sedimentation rate (ESR), C-reactive protein (CRP), and hematocrit], disease activity assessed by Crohn's Disease Activity Index (CDAI) score, the change in treatment regimen, and the utilization of telemedicine, were retrospectively collected for each patient by 2 independent observers. Baseline data were recorded at the last treatment before the COVID-19 outbreak. The follow-up was performed by telephone or internet. The Pittsburgh Sleep Quality Index (PSQI) was used to evaluate the quality of patients' sleep (19). The need for surgery and readmission during the follow-up period were identified as indicators of the long-term influence.

### Definition

Treatment delay was defined as more than 2 weeks after the scheduled date. This study chose 2 weeks in light of logistical challenges regarding previous studies (20). The deadline for follow-up was December 31, 2020. The secondary endpoint was the occurrence of a surgical or readmission event during follow-up. The time of first infliximab exposure during the outbreak was regarded as the time point of short-term efficacy assessment. Patients who underwent gastrointestinal surgery excluding anal fistula during the follow-up were considered to have a surgical history. A history of readmission was defined as readmission due to recurrence or exacerbation of the disease with a hospital stay longer than 2 days.

### Statistical Analyses

Continuous variables expressed as medians with interquartile ranges (IQRs) or means ± standard deviations (SDs) depending on the underlying data distribution were used to describe the statistics of the cohort. Independent Student's t-test and Wilcoxon rank-sum test were used where appropriate. Categorical variables were described as numbers with percentages. Pearson's chi-squared test was used for independent categorical variables. Univariate analysis was performed to identify the baseline variables that were significantly different between the delayed and non-delayed groups. Multivariable logistic regression analyses were performed to determine associations with short-term influence and baseline characteristics. The factors with *p* < 0.1 in univariate analysis were included in multivariate analysis. A Kaplan-Meier curve was used to evaluate the surgical or readmission events. Propensity score matching (PSM) was applied in this study to increase the comparability between the two groups. A binary logistic regression test was performed to generate a propensity score for each patient with and without delayed treatment by the significant factors. Subsequently, a one-to-one match between both groups was obtained using the nearest-neighbor matching method with a Caliper value of 0.2 (21). All reported *P-*values are two-tailed, and *p* < 0.05 indicated statistical significance. Analyses were performed with SPSS Statistics version 26.0 software.

### Ethical Considerations

The study was approved by the Xiangya Hospital of Central South University Ethics Committees, and each subject provided written, informed consent prior to study participation.

## Results

### Characteristics of the Study Population

**Figure 1:**
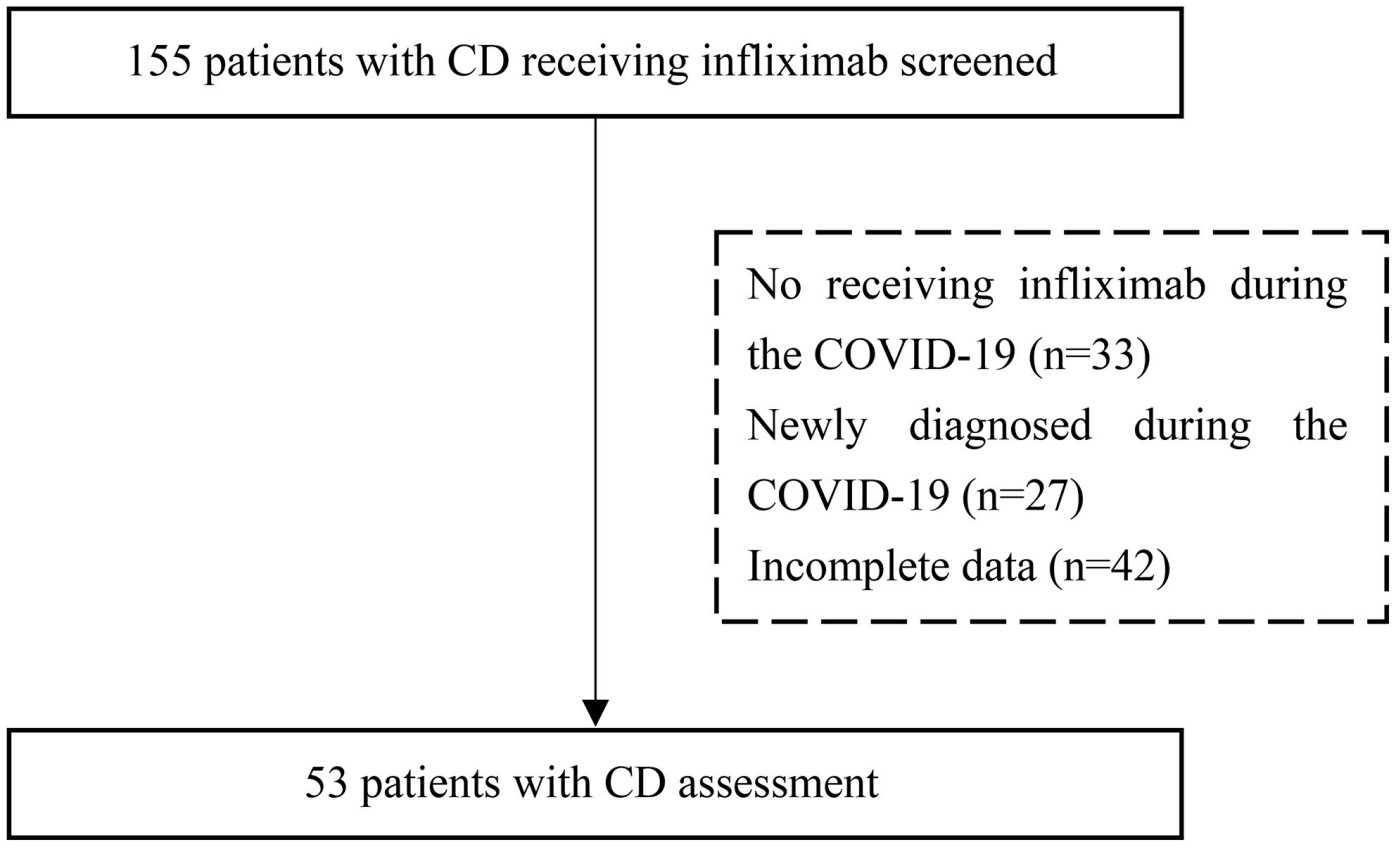
A flow chart of the inclusion process for patients with Crohn's disease who were treated with infliximab. CD, Crohn's disease.

A total of 155 patients with Crohn's disease were included in this study. A schematic diagram of the selection process is shown in Figure 1. Fifty-three patients met the inclusion criteria. The patients included 34 (64.2%) males and 19 (35.8%) females with a median age of 26.0 years (IQR 18.5–36.0). Approximately three-quarters (71.7%) of patients did not receive treatment as planned with an average delay of 43.1 ± 28.3 days.

Table 1 presents the baseline data between both groups. Patients in the delayed group had significantly lower CDAI scores [89.7 (58.5, 116.9) vs. 125.9 (99.0, 200.8), *p* = 0.030] and C-reactive protein (CRP) levels [3.8 (1.5, 12.1) vs. 8.9 (2.0, 39.4), *p* = 0.030] than those in the non-delayed group. No differences in sex or age were noted between the groups. Disease duration, other serological indicators, and the utilization of telemedicine at baseline were similar in the delayed group compared with the non-delayed group (*p* > 0.05).

**Table 1:** Baseline characteristic of the enrolled patients.

**Variable**	**Total patients (*n* = 53)**	**Delayed group (*n* = 38)**	**Non-delayed group (*n* = 15)**	P^*^
Male, No. (%)	34.0 (64.2)	23.0 (60.5)	11.0 (73.3)	0.381
Age, median (IQR), year	26.0 (18.5, 36.0)	27.0 (19.0, 38.0)	24.0 (16.8, 32.3)	0.243
Height, median (IQR), cm	167.0 (160.0, 170.0)	165.0 (160.0, 170.0)	170.0 (165.3, 172.5)	0.052
Weight, median (IQR), kg	52.5 (48.0, 60.0)	53.0 (48.0, 60.0)	51.3 (43.8, 63.0)	0.901
BMI, median (IQR), kg/m^2^	18.9 (17.6, 21.1)	19.0 (17.7, 21.3)	18.5 (16.2, 21.1)	0.343
Disease course, median (IQR), year	2.0 (1.0, 5.0)	3.0 (1.0, 5.0)	1.0 (0.7, 3.9)	0.051
CDAI, median (IQR)	96.6 (60.1, 144.0)	89.7 (58.5, 116.9)	125.9 (99.0, 200.8)	0.030
Utilization of telemedicine, No. (%)	26.0 (49.1)	21.0 (55.3)	5.0 (33.3)	0.150
Serological parameters				
ESR, median (IQR), mm/h	34.0 (18.5, 62.5)	34.0 (19.5, 54.3)	38.0 (15.5, 77.5)	0.657
CRP, median (IQR), mg/L	4.4 (1.6, 15.2)	3.8 (1.5, 12.1)	8.9 (2.0, 39.4)	0.030
Hematocrit, median (IQR), %	38.6 (22.6, 42.1)	39.4 (36.5, 44.5)	40.6 (36.6, 44.6)	0.778

*BMI, body mass index; CDAI, Crohn's Disease Activity Index score; ESR, erythrocyte sedimentation rate; CRP, C-reactive protein; IQR, interquartile range.*

* *Mann-Whitney U test for continuous variables and chi-square for proportions*.

### Comparison of the Delayed and Non-delayed Groups

On univariable analysis, the baseline characteristics that predicted delayed treatment during the COVID-19 epidemic included lower CDAI scores (OR = 0.0991, 95% CI = 0.982–1.000, *p* = 0.05) and lower CRP levels (OR = 0.965, 95% CI = 0.933–0.998, *p* = 0.038), as presented in Table 2. Additionally, lower CRP levels (OR = 0.967, 95% CI = 0.935–1.000, *p* = 0.047) were the only significant predictor of a delayed infusion of infliximab for CD patients in the multivariable analysis.

**Table 2:** Univariable and multivariable analyses of risk factors of delayed treatment.

**Variable**	**Univariate analysis**		**Multivariate analysis**	
	**HR (95%CI)**	***p***	**HR (95%CI)**	***p***
Male	0.392 (0.094–1.634)	0.198		
Age, year	1.040 (0.980–1.103)	0.192		
Height, cm	0.904 (0.810–1.008)	0.071		
Weight, kg	1.000 (0.933–1.072)	0.998		
BMI, kg/m^2^	1.131 (0.900–1.421)	0.29		
Disease course, year	1.241 (0.940–1.639)	0.127		
CDAI	0.991 (0.982–1.000)	0.05		
Utilization of telemedicine	0.476 (0.135–1.681)	0.476		
Serological parameters				
ESR, mm/h	0.993 (0.975–1.012)	0.462		
CRP, mg/L	0.965 (0.933–0.998)	0.038	0.967 (0.935–1.000)	0.047
Hematocrit, %	1.023 (0.927–1.128)	0.652		

*BMI, body mass index; CDAI, Crohn's Disease Activity Index score; ESR, erythrocyte sedimentation rate; CRP, C-reactive protein; HR, hazard ratio; CI, confidence interval*.

### Characteristics of the Study Population After Propensity Score-Matched Analysis

To circumvent covariate imbalance between the two groups, a 1:1 propensity score-matched analysis was performed, resulting in 15 patients in the delayed group being matched with 15 non-delayed patients. There were no significant differences in the baseline CDAI score [135.6 (94.1, 185.2) vs. 125.9 (99.0, 200.8), *p* = 1.000] and CRP levels [10.4 (2.4, 21.5) vs. 8.9 (2.0, 39.4), *p* = 0.540] after matching, nor in the remaining variables (*p* > 0.05). The baseline characteristics after propensity score matching are indicated in Table 3.

**Table 3:** Baseline characteristic of the enrolled patients after propensity score-matched analysis.

**Variable**	**Total patients (*n* = 30)**	**Delayed group (*n* = 15)**	**Non-delayed group (*n* = 15)**	P^*^
Male, No. (%)	22.0 (73.3)	11.0 (73.3)	11.0 (73.3)	1.000
Age, median (IQR), year	24.0 (17.8, 33.8)	23.5 (18.0, 35.3)	24.0 (16.8, 32.3)	0.697
Height, median (IQR), cm	170.0 (160.0, 172.0)	165.0 (160.0, 170.0)	170.0 (165.3, 172.5)	0.131
Weight, median (IQR), kg	50.5 (44.8, 57.8)	50.5 (45.6, 55.1)	51.3 (43.8, 63.0)	0.473
BMI, median (IQR), kg/m^2^	18.3 (16.2, 21.1)	18.3 (15.9, 21.2)	18.5 (16.2, 21.1)	0.822
Disease course, median (IQR), year	2.0 (1.0, 4.3)	3.0 (1.5, 4.5)	1.0 (0.7, 3.9)	0.093
CDAI, median (IQR)	130.4 (101.9, 188.9)	135.6 (94.1, 185.2)	125.9 (99.0, 200.8)	1.000
Utilization of telemedicine, No. (%)	12.0 (40.0)	7.0 (46.7)	5.0 (33.3)	0.456
Serological parameters				
ESR, median (IQR), mm/h	42.5 (23.0, 76.5)	45.0 (33.3, 74.3)	38.0 (15.5, 77.5)	0.667
CRP, median (IQR), mg/L	9.2 (2.1, 25.5)	10.4 (2.4, 21.5)	8.9 (2.0, 39.4)	0.608
Hematocrit, median (IQR), %	39.2 (36.4, 44.4)	39.1 (36.3, 41.4)	40.6 (36.6, 44.6)	0.377

*BMI, body mass index; CDAI, Crohn's Disease Activity Index score; ESR, erythrocyte sedimentation rate; CRP, C-reactive protein; IQR, interquartile range.*

* *Mann-Whitney U-test for continuous variables and chi-square for proportions*.

### Short- and Long-Term Effects of Delayed Treatment

On the short-term efficacy assessment, data after matching for details reported in Table 4 indicated that patients in the non-delayed group experienced a greater decrease in CDAI score compared to the delayed group [−43.3 (−92.7, −9.7) vs. −17.3 (−29.0, 79.9), *p* = 0.038]. Delayed treatment appeared to have minimal impacts on ESR levels, CRP levels, hematocrit levels, PSQI score, and change in clinical decision making, whereas trends toward increased weight and BMI were noted in the delayed group (*p* = 0.070).

**Table 4:** The comparison of the effect of delayed treatment between both groups after propensity score-matched analysis.

**Variable**	**Total patients (*n* = 30)**	**Delayed group (*n* = 15)**	**Non-delayed group (*n* = 15)**	P^*^
Short-term effect				
Weight, median (IQR), kg	53.5 (46.4, 60.4)	53.5 (44.3, 57.3)	52.5 (46.3, 62.4)	0.759
ΔWeight, median (IQR), kg	0 (−1.5, 3.3)	1.5 (−0.4, 4.0)	−0.8 (−1.9, 0.6)	0.070
BMI, median (IQR), kg/m^2^	18.8 (16.3, 21.4)	18.9 (16.1, 22.5)	18.7 (16.3, 21.2)	0.697
ΔBMI, median (IQR), kg/m^2^	0 (−0.5, 1.2)	0.5 (−0.1, 1.4)	−0.3 (−0.7, 0.2)	0.070
CDAI, median (IQR)	103.7 (79.4, 185.2)	135.8 (85.4, 235.5)	91.7 (67.9, 121.9)	0.093
ΔCDAI, median (IQR)	−27.1 (−70.8, 34.4)	−17.3 (−29.0, 79.9)	−43.4 (−92.7, −9.7)	0.038
Change in clinical decision making, No. (%)	9.0 (30.0)	5.0 (16.7)	4.0 (13.3)	0.69
PSQI, median (IQR)	4.0 (3.0, 5.5)	4.5 (4.0, 6.8)	4.0 (2.0, 5.0)	0.156
Serological parameters				
ESR, median (IQR), mm/h	40.0 (17.0, 68.0)	35.0 (18.3, 73.3)	42.0 (12.3, 61.8)	0.886
ΔESR, median (IQR), mm/h	−2.0 (−23.3, 10.8)	−2.0 (−26.8, 8.0)	−4.5 (−22.5, 19.5)	0.667
CRP, median (IQR), mg/L	9.1 (2.0, 23.9)	10.2 (1.8, 36.0)	9.1 (5.6, 16.8)	1.000
ΔCRP, median (IQR), mg/L	0.6 (−2.4, 9.2)	1.0 (−2.1, 12.3)	−4.5 (−22.5, 19.5)	0.313
Hematocrit, median (IQR), %	40.1 (34.7, 44.2)	40 (34.9, 43.7)	41.4 (33.9, 44.8)	0.728
ΔHematocrit, median (IQR), %	1.6 (−3.4, 3.4)	1.0 (−1.9, 3.3)	2.2 (−4.5, 4.1)	0.951
Long–term effect				
Surgery, No. (%)	3 (10.0)	3 (20.0)	0 (0.0)	0.068
Readmission, No. (%)	5 (16.7)	5 (33.3)	0 (0.0)	0.014

*BMI, body mass index; CDAI, Crohn's Disease Activity Index score; PSQI, Pittsburgh sleeps quality index score; ESR, erythrocyte sedimentation rate; CRP, C-reactive protein; IQR, interquartile range; Δ, the difference between the recent value of the variable and the baseline value.*

* *Mann-Whitney U test for continuous variables and chi-square for proportions*.

**Figure 2:**
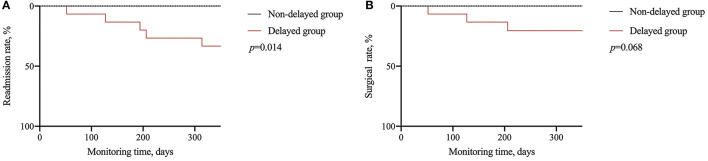
Long-term effect of delayed treatment. **(A)** Comparison of readmission rate; **(B)** comparison of surgical rate.

Considering the long-term influence, the continuous follow-up of enrolled patients after matching showed that the delayers had a higher readmission rate (33.3% vs. 0%, *p* = 0.014) and a higher surgery rate, although the difference was not statistically significant (*p* = 0.068). Outcomes evaluated for each endpoint are displayed in Table 4 and Figure 2.

## Discussion

The advent of biologics has presented new options for the treatment of inflammatory bowel disease, and infliximab is the most widely used biological agent in the clinic (22). However, there are issues with the required intravenous injection, especially with nonhuman biological agents that are prone to allergic reactions; specifically, patients have to physically come to the hospital for infusion (23). Due to the COVID-19 outbreak, patients opted to decrease their chances of contracting COVID-19 by providing home self-care rather than going to the hospital for regular care, which has caused a greater than 50% reduction in hospitalization volume (24). On the other hand, whether infliximab increased the risk of viral infection remained ambivalent early in the epidemic (25, 26). Above all, Crohn's disease patients experienced great difficulty in adhering to the infliximab schedule. In addition, the prognostic impact of a short delay in treatment on Crohn's disease remains unknown.

In this study, the final cohort of 53 patients with Crohn's disease previously treated with standard infliximab demonstrated a delayed rate of 71.7%, which is considerably increased compared with 26.4% in Khan's study (20). This finding may be related to policies in different countries at the beginning of the epidemic as well as the population's sense of self-protection. Additionally, this study suggested that delayed treatment was significantly associated with the CDAI score and CRP level at baseline. CRP is an acute-phase protein that increases with the aggravation of inflammatory reactions (27) and can also objectively respond to disease activity in CD patients (28). Therefore, the reason for this effect might be attributed to the fact that the more severe disease patients have, the more the disease is actively treated.

For the short-term effects, this study found that delayed treatment with infliximab led to a lower decrease in the CDAI score, suggesting worse control of symptoms in these patients, but this finding is not consistent with the results of serological indicators, such as CRP and ESR. However, these indicators are not specific markers of intestinal inflammation; other factors (such as infection or extraintestinal inflammation) can also affect the results (29). Indicators, such as endoscopic activity score and fecal calprotectin, should be included in further studies. For long-term prognosis, this study identified that delayed treatment with infliximab increased the readmission and operation rates of patients, which may be due to the lower infliximab concentrations in blood. Previous studies have shown that the interval of administration was closely related to the blood infliximab concentration and treatment effects. During maintenance treatment of IBD patients, the trough concentrations of infliximab in patients treated with a 4-week interval was significantly higher than that of patients treated with an 8-week interval (30). The trough concentration of infliximab was positively correlated with the remission rate of IBD (31). In addition, Scaldaferri et al. found that the noncompliance of patients during infliximab treatment could significantly increase the concentration of the antibody in serum and the rate of secondary treatment failure (32). Khan's study also showed that irregular infusion of biological agents was significantly associated with the rate of glucocorticoid-free remission during the COVID-19 epidemic (20).

There are some innovations in the study. First, previous studies mostly focused on the impact of long-term irregular delayed infliximab treatment on the prognosis of patients with Crohn's disease (33, 34), whereas this study showed that the short-term delay in infliximab treatment during the COVID-19 pandemic may also affect the long-term prognosis of patients. Second, the propensity matching method was used to eliminate possible confounding factors, thus increasing the comparability between the two groups of patients and making the results more reliable. However, this study is a single-center retrospective study with a small sample size, which needed for further verification.

## Conclusions

In conclusion, the study suggested that approximately three-quarters of CD patients receiving infliximab regularly have delays in their treatment during the COVID-19 epidemic, thereby affecting which the independent risk factor was CRP levels at baseline. Delayed treatment may not only aggravate short-term disease activity but also increase the readmission rate in the future. Therefore, implementation of infliximab infusion based on an effective schedule and education programs for patients about the relative safety of these biologic medications are needed for patients and clinicians despite the COVID-19 pandemic.

## Data Availability Statement

The raw data supporting the conclusions of this article will be made available by the authors, without undue reservation.

## Ethics Statement

The study was approved by the Xiangya Hospital of Central South University Ethics Committees, and each subject provided written, informed consent prior to study participation. The patients/participants provided their written informed consent to participate in this study.

## Author Contributions

YL, LC, SC, and XL: study concept and design and critical revision of the manuscript for important intellectual content. SC and LC: acquisition of data. XL and YL: analysis and interpretation of data. YL and LC: statistical analysis and drafting of the manuscript. All authors approved the final version of the manuscript, including the authorship list.

## Funding

This work was supported by the National Natural Science Foundation of China (Grant Nos. 81770584 and 81700464) and Natural Science Foundation of Hunan Province (No. 2018JJ3841).

## Conflict of Interest

The authors declare that the research was conducted in the absence of any commercial or financial relationships that could be construed as a potential conflict of interest.

## Publisher's Note

All claims expressed in this article are solely those of the authors and do not necessarily represent those of their affiliated organizations, or those of the publisher, the editors and the reviewers. Any product that may be evaluated in this article, or claim that may be made by its manufacturer, is not guaranteed or endorsed by the publisher.
